# Green Synthesis of Coal Gangue-Derived NaX Zeolite for Enhanced Adsorption of Cu^2+^ and CO_2_

**DOI:** 10.3390/ma18071443

**Published:** 2025-03-25

**Authors:** Yanshuang Chen, Yilin Chen, Hanqi Xu, Wenqi Zhao, Guodong Feng, Chunhui Xiao

**Affiliations:** Engineering Research Center of Energy Storage Materials and Devices, Ministry of Education, School of Chemistry, Xi’an Jiaotong University, Xi’an 710049, China; chenyanshuang@stu.xjtu.edu.cn (Y.C.); chenyl123@stu.xjtu.edu.cn (Y.C.); xhq23@stu.xjtu.edu.cn (H.X.); zwqaqi@stu.xjtu.edu.cn (W.Z.)

**Keywords:** coal gangue valorization, Alkali fusion–hydrothermal synthesis, NaX zeolite, heavy metal adsorption, CO_2_ capture

## Abstract

The accumulation of coal gangue (CG), a byproduct of coal mining, poses severe environmental challenges. This study presents a green strategy to convert CG into high-value NaX zeolite via an alkali fusion–hydrothermal method. Through orthogonal experiments, the optimal synthesis conditions (solid–liquid ratio 1:8, crystallization temperature 110 °C, time 12 h) were identified, yielding NaX zeolite with exceptional crystallinity (98%), specific surface area (703.5 m^2^/g), and pore volume (0.28 cm^3^/g). Comprehensive characterization (XRD, SEM-EDS, BET, etc.) confirmed its structural integrity and thermal stability. The synthesized zeolite exhibited remarkable adsorption capacities for Cu^2+^ (185.35 mg/g) and CO_2_ (5.51 mmol/g), following the Langmuir isotherm model. This work not only addresses gangue disposal challenges but also demonstrates a cost-effective route for producing high-performance adsorbents, aligning with circular economy and carbon neutrality goals.

## 1. Introduction

**Figure 1:**
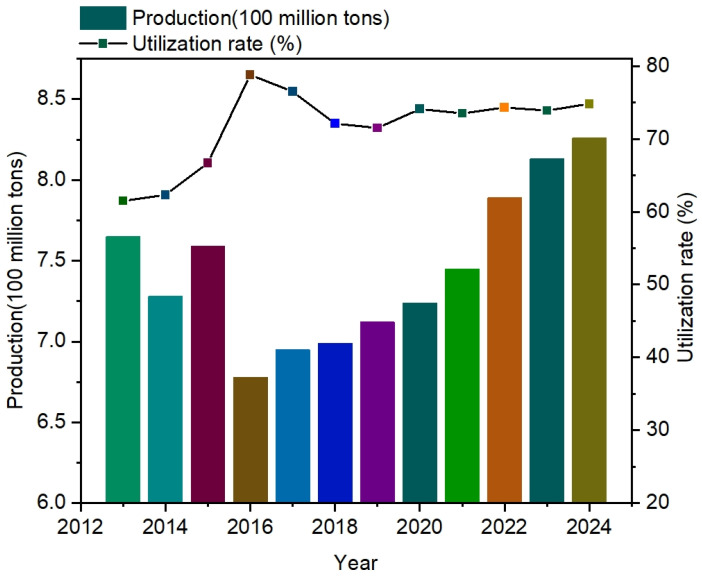
Visualization of gangue production and utilization in recent years.

Coal gangue (CG) is a type of solid waste associated with coal mining operations [1,2]. As shown in Figure 1, coal gangue production has increased annually with the continuous development of the coal industry [3,4]. However, comprehensive survey data for its utilization rate are lacking. As a result, coal gangue from the process of coal mining is piled up in large quantities, causing serious environmental pollution and natural disasters. It accounts for more than 40% of the solid waste generated in the mining sector, and it has been listed as one of the key concerns of the Outline of the Fourteenth Five-Year Plan for the National Economic and Social Development of the People’s Republic of China and Vision 2035. At present, driven by the goal of “carbon peak and carbon neutrality”, coal gangue dumping is receiving increasing attention [5]. The problem of coal gangue dumping in various mining areas is mainly reflected in the large area of dumping, high environmental pollution, difficulty of comprehensive utilization, and so on. Although there are many cases of comprehensive utilization of coal gangue [6], the comprehensive utilization rate of coal gangue is still less than 60%, and a more standardized and feasible research system for the comprehensive utilization and disposal of coal gangue has not yet been formed [7]. Most applications are basic, such as backfilling and road construction; additionally, the economic benefits are very low, presenting high labor costs, so there is no clear approach with which to solve the problem of pollution caused by coal gangue.

NaX-type molecular sieves are a class of porous three-dimensional silicate–aluminate crystals [8,9]. The crystal unit is formed by silica–oxygen tetrahedra and aluminum–oxygen tetrahedra through oxygen atom bridging and a three-dimensional cage structure [10]. These molecular sieves can adsorb molecules that are smaller than their own pore size [11] while larger diameter molecules are excluded. Because NaX-type molecular sieves have a microporous and void structure inside, they have high specific surface area (300–1000 m^2^/g) and rich pore capacity [12,13]. These characteristics make their application in the field of adsorption very significant.

According to previous research, the main components of gangue are Al_2_O_3_ and SiO_2_, as well as a small amount of carbon-containing organic matter and other metal oxides, which are the main raw materials for the synthesis of silica–aluminum-based porous molecular sieves [14,15]. Impurities can be removed from the gangue through the pre-treatment stage, with full extraction of the effective silica–aluminum elements, so as to use the gangue to prepare gangue-based NaX-type porous molecular sieves with high surface area, large pore volume, and high adsorption performance [16]. Adsorption treatments for waste water and waste gas, especially those produced in the process of coal chemical industry operations, can effectively reduce environmental pollution and reduce emissions of waste water and waste gas from enterprises [17,18]. This provides the potential to solve the problem of coal gangue dumping and pollution, while also achieving the goal of converting coal gangue “waste into treasure, waste to waste”. Therefore, the synthesis of NaX-type molecular sieves with gangue as a raw material for three-waste treatment can effectively solve the double challenge of the recovery and treatment of coal gangue. In environmental remediation engineering, an X-type molecular sieve material synthesized from coal gangue is developed as a highly efficient adsorbent compound. This material specifically captures nutrient salt pollutants such as ammonium nitrogen and phosphate in water bodies through the ion exchange mechanism. At the same time, it can effectively retain a variety of heavy metal ions, such as Pb, Cd, Hg, etc., which is of practical significance for the control of the eutrophication of water bodies and heavy metal pollution. Ge et al. synthesized Na-X molecular sieves from coal gangue powder (CGP) using an alkali-fusion hydrothermal method, achieving a maximum Pb^2+^ adsorption capacity of 457 mg/g [19].

Coal will remain China’s primary energy source for the foreseeable future. However, rapid industrial development has exacerbated environmental challenges, necessitating urgent management of coal-derived solid waste [20,21]. Solving the problem of a large number of coal gangue piles and finding suitable adsorbents to deal with heavy metal pollution of wastewater from the coal chemical industry, as well as reducing greenhouse gas CO_2_ emissions, have become the main challenges of the dual-carbon background [22,23]. Recent years have seen preliminary progress in coal gangue management through technological innovation, policy regulation, and regional practices. For instance, Qitaihe City established a coal gangue utilization park producing high-value derivatives (e.g., kaolin, ceramics, and composite fertilizers), annually consuming 5 million tons of gangue. Collaborations with universities and provincial research centers have fostered a “resource-product-waste-renewable resources” industrial chain [24,25], Nevertheless, current utilization efficiency remains suboptimal [26,27], with most applications confined to low-value primary stages. To address this gap, this study synthesizes NaX-type porous molecular sieves for high-value applications, including heavy metal ion/macromolecule adsorption in wastewater [28,29], CO_2_ capture [30,31,32], and cost-effective molecular sieve production [33]. This strategy enables multi-perspective resource valorization of coal gangue [34,35], offering innovative pathways for sustainable recycling.

This research aims to utilize bulk coal gangue solid waste as a raw material for the production of functional porous X-type molecular sieves via alkali-fusion hydrothermal synthesis. The molecular sieves are designed for dual applications: heavy metal (Cu^2+^) removal from wastewater and CO_2_ capture, achieving a “waste-to-resource” strategy. Key investigations include (1) the optimization of gangue pretreatment and structural reconstruction, (2) directional synthesis and performance modulation of gangue-based porous molecular sieves, and (3) the establishment of process–property relationships using XRD, SEM, and BET analyses. These efforts enable precise regulation of the surface chemistry, pore topology, and adsorption capacity of the synthesized materials.

## 2. Experimental Materials and Contents

### 2.1. Materials

The gangue used for the experiments was obtained from Wo Cao Gou No. 2 Coal Mine Co., Ltd. (Yan’an City, Shaanxi Province, China). Only the gangue was used to provide an adequate source of silicon and some aluminum. Concentrated hydrochloric acid (HCl, AR 36~38 vt%) was obtained from Huttest (Tianjin, China), sodium hydroxide (NaOH, AR) from McLean Biochemical Technology Co., Ltd. (Shanghai, China), sodium meta-aluminate (NaAlO_2_, AR) and copper sulfate pentahydrate (CuSO_4_-5H_2_O, AR) from Aladdin Reagent Co., Ltd. (Shanghai, China), and carbon dioxide gas (CO_2_, high purity) from Lietzel Gas Co., Ltd. (Beijing, China)gas. Deionized water (H_2_O, 18.25 MΩ·cm) was made in our laboratory.

### 2.2. Preparation Method of Gangue-Based NaX-Type Molecular Sieve

The gangue samples are primarily composed of inert amorphous quartz and kaolinite crystalline structures, requiring pretreatment processes including crushing and calcination to remove carbon and organic impurities. Furthermore, the SiO_2_/Al_2_O_3_ ratio in raw gangue does not precisely match the synthesis requirements of NaX-type molecular sieves, necessitating supplementation with an external aluminum source to achieve the crystalline phase range of NaX molecular sieves. In this study, NaX-type molecular sieves were synthesized via a one-step alkali-fusion hydrothermal method, wherein sodium hydroxide served dual functions: providing sodium ions and maintaining the pH environment required for nucleation.

The experimental workflow proceeded as follows: Raw gangue underwent coarse pretreatment involving crushing and calcination to disrupt dense inert components and remove iron impurities. The calcined material was then ball-milled (200 rpm) for 180 min, sieved to 200–400 mesh, and dried. To ensure experimental consistency, a bulk batch of gangue powder was prepared by calcining at 750 °C for 2 h, followed by acid leaching with 4 mol/L hydrochloric acid at a solid-to-liquid mass ratio of 1:15. This process was conducted at 65 °C for 12 h under 300 rpm magnetic stirring. The leached material was subsequently washed to neutrality, oven-dried at 60 °C, and characterized via XRF to quantify Si/Al ratios and impurity removal rates. All subsequent experiments utilized this standardized acid-leached gangue batch.

Alkali fusion–hydrothermal crystallization: For experimental standardization, 6 g of acid-leached gangue and 7.2 g of sodium hydroxide powder were weighed and homogenized, followed by alkali fusion in a muffle furnace at 650 °C for 2 h (heating rate: 10°C/min). Post-fusion, the mixture was transferred to a conical flask, mixed with 105 mL deionized water, and stirred to form a homogeneous suspension. The critical SiO_2_/Al_2_O_3_ ratio was then adjusted to 1.1–1.65 (molar ratio), maintaining the alkaline solution concentration at ~2.5 mol/L. Based on stoichiometric calculations, 2.4 g of sodium aluminate (NaAlO_2_) was added to supplement the aluminum source, ensuring the ratio aligned with the NaX molecular sieve crystalline phase region. The mixture was aged for 12 h to disrupt the native gangue structure, enabling SiO_4_ and AlO_4_ tetrahedra rearrangement into crystalline precursors and initial framework formation.

**Figure 2:**
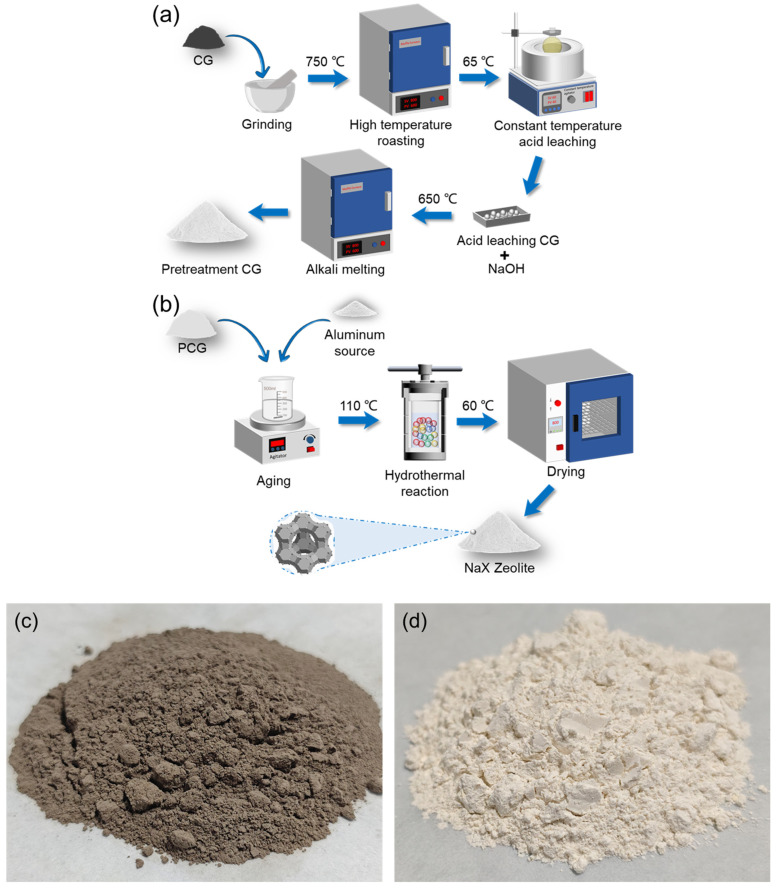
(**a**) Acid leaching treatment process, (**b**) Alkali fusion-hydrothermal reaction, (**c**) Coal gangue, (**d**) NaX zeolite.

The aged slurry was transferred to a PTFE-lined autoclave for hydrothermal crystallization (110 °C, 12 h). Under elevated temperature and pressure, SiO_4_ and AlO_4_ units assembled around nucleation sites, ultimately forming the molecular sieve framework. Post-crystallization, the product was centrifuged (8000 rpm, 5 min), washed to near neutrality, and dried at 60 °C. Figure 2 illustrates the synthesis workflow.

### 2.3. Orthogonal Experimental Design

Orthogonal experimental design is a multi-factor and multi-level optimization method that leverages orthogonality to select representative test points from the full parameter space. These points exhibit “uniform dispersion and comparability”, enabling efficient, rapid, and economical exploration of variable interactions. In this study, given the multiple factors influencing NaX-type molecular sieve synthesis, a three-factor (solid-to-liquid ratio, crystallization time, crystallization temperature) and three-level orthogonal design was implemented. Table 1 shows 9 orthogonal experiments in detail. Product performance was evaluated via specific surface area measurements.

The factor selection aligns with the three-stage kinetic theory of zeolite hydrothermal synthesis:

(1) Solid-to-liquid ratio: Governs system alkalinity (OH^−^ concentration) and silica-alumina precursor solubility, critically influencing nucleation rates (literature-reported correlation coefficient between this ratio and crystallinity: R > 0.8);

(2) Crystallization temperature: Modulates activation energy as per the Arrhenius equation, where a 10 °C increase elevates crystallization rates 2–3-fold (activation energy E_a_ ≈ 45 kJ/mol);

(3) Crystallization time: Balances crystal growth integrity (insufficient duration induces amorphous impurities) against over-crystallization risk (e.g., phase transitions or grain coarsening). These parameters collectively control nucleation-growth equilibria and are established control variables in molecular sieve synthesis optimization.

### 2.4. Adsorption Experiment Design

To evaluate the heavy metal ion adsorption capacity of NaX-type molecular sieves, simulated copper-laden wastewater was treated with the material. The Cu^2+^ concentration variation was quantified via UV-V is spectrophotometry (400–600 nm wavelength range) by measuring absorbance changes pre- and post-adsorption under controlled laboratory conditions.

For CO_2_ adsorption analysis, approximately 400 mg of activated molecular sieves was degassed at 250 °C under vacuum for 8 h. After cooling, the sample mass was recorded in BET analysis software (Micromeritics ASAP 2460 Version 3.01.02). The activated material was then loaded into a reaction vessel, immersed in a 0 °C ice-water bath, and purged with helium at 40 mL/min. Concurrently, CO_2_ gas was introduced at 50 mL/min for 10 h. Adsorption isotherms were generated by analyzing CO_2_ uptake at 0 °C and 25 °C under varying pressures using the BET system.

### 2.5. Characterization

This study employed a multi-dimensional characterization system to analyze the physicochemical properties of gangue-derived X-type molecular sieves:

(1) X-ray fluorescence spectroscopy (XRF): Utilizing characteristic X-ray excitation principles, elemental compositions (Si, Al, Fe) were quantified (accuracy: ±0.5%) by detecting fluorescence intensities in raw and acid-modified gangue, establishing the SiO_2_/Al_2_O_3_ ratios for synthesis process optimization.

(2) Thermogravimetric analysis (TGA): Thermogravimetric-differential thermal coupling tracked mass loss from 20–1000 °C (heating rate: 10 °C/min), identifying organic decomposition and carbonate removal events to determine optimal calcination temperatures while avoiding component degradation.

(3) X-ray diffraction (XRD): Bragg’s law-based analysis with Cu-Kα radiation (λ = 0.154 nm) generated 0°–90° diffraction patterns (scan rate: 5°/min). Phase identification via ICDD PDF#38-0237 and crystallinity calculations using the Scherrer equation confirmed crystallization completeness.

(4) Fourier transform infrared spectroscopy (FT-IR): Molecular vibration modes (400–4000 cm^−1^) revealed T-O-T bending vibrations and bicyclic structural signatures, verifying framework formation and chemical bonding states.

(5) Scanning electron microscopy-energy dispersive spectroscopy (SEM-EDS): Secondary electron imaging (5 kV, 5000×–20,000×) visualized cubic morphologies, while EDS mapping validated homogeneous Si/Al distributions.

(6) Transmission electron microscopy (TEM): High-resolution imaging (200 kV) resolved 0.5 nm lattice fringes ((111) crystal planes), with selected area electron diffraction (SAED) confirming cubic symmetry. Elemental mapping ensured Si/Al ratio consistency with XRF data.

(7) Brunauer-Emmett-Teller (BET) analysis: N_2_ adsorption/desorption at 77 K (−196 °C) yielded specific surface areas, pore size distributions (BJH model), and total pore volumes, systematically evaluating adsorption capacities and mass transfer properties.

Collectively, these techniques establish a holistic analytical framework spanning chemical composition, thermal behavior, crystallinity, molecular vibrations, micromorphology, and porosity, enabling multi-scale synthesis optimization and performance assessment of gangue-based molecular sieves.

## 3. Results and Discussion

### 3.1. Coal Gangue Raw Material and Pretreatment Analysis

XRF analysis revealed that the experimental gangue predominantly comprised SiO_2_ and Al_2_O_3_ (combined content >85%), with trace metal impurities including Fe, Mg, Mn, Cr, Cu, Pb, Ti, Co, Ni, Zn, and Zr. The detailed composition is shown in Table 2. Among these, iron emerged as the principal contaminant, necessitating chemical removal during pretreatment to minimize interference with alkali fusion and ensure zeolite purity. The raw gangue exhibited a molar SiO_2_/Al_2_O_3_ ratio of 1.57, aligning with the optimal range for synthesizing low-silica-alumina-ratio zeolitic molecular sieves.

**Figure 3:**
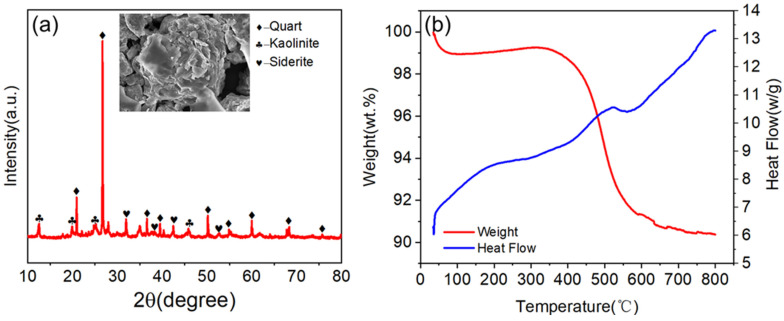
(**a**) XRD pattern of gangue. (**b**) TG−DSC curve of gangue.

The XRD pattern of the raw gangue confirmed quartz, kaolinite, and rhodochrosite as its primary phases. SEM imaging further revealed a rough, heterogeneous microstructure dominated by lamellar disordered morphologies. Thermogravimetric (TGA) analysis exhibited three distinct mass loss stages: (1) 0–100 °C (moisture and humus removal), (2) 400–750 °C with peak mass loss at 480 °C (carbon combustion), and (3) >750 °C (stable residual mass). The negligible weight loss beyond 750 °C indicated complete elimination of combustible impurities, validating the 750 °C activation temperature as optimal. Figure 3 shows the detailed test results.

The experimental results demonstrate that raw gangue cannot be directly utilized as experimental feedstock but requires thermal activation via high-temperature calcination in a muffle furnace to remove carbon, organic matter, and other impurities. Subsequent acid leaching with 4 mol/L hydrochloric acid effectively eliminated iron and magnesium contaminants. Table 3 quantitatively compares the compositional changes of gangue before and after acid leaching.

Table 3 data show that after acid leaching treatment, the relative content of SiO_2_ increased, while the content of Al_2_O_3_ decreased, and the Fe_2_O_3_/MgO content was reduced to less than one percent, which indicated that the acid leaching treatment effect was appropriate, i.e., the soluble metal oxides had been effectively removed. Although the acid leaching process lost some of the Al^3+^, a significant amount of effective aluminum was retained.

Acid leaching to remove Fe impurities can effectively reduce their damage to the alkali melting process. This can be explained as follows.

(1) The oxidation state of Fe regulates the kinetics of silica-alumina dissolution: under strong alkaline melting conditions (NaOH/gangue > 0.3), Fe^2+^ acts as a mineralizer and promotes the breaking of Si-O and Al-O bonds through liganding, accelerating the dissolution of silica-alumina precursor (activation energy reduced by ~15%). Fe^3+^ tends to form colloidal Fe(OH)_3_ (precipitated at pH > 12), which encapsulates unreacted SiO_2_/Al_2_O_3_ particles, thus inhibiting the release of silica-alumina monomers and leading to a decrease in crystallinity. (2) Interference of Fe doping on crystal growth: Fe^3+^ (radius 0.64 Å) may partially replace Al^3+^ (0.53 Å) into the zeolite skeleton, which triggers local lattice distortions and reduces the molecular sieves’ thermal stability. Moreover, Fe^2+^/Fe^3+^ cycle catalyzes the generation of hydroxyl radicals (OH), which triggers the de-alumination of the skeleton and disrupts the structural order of the zeolite.

### 3.2. Analysis of Experimental Results of Orthogonal Synthesis of NaX-Type Molecular Sieves

This study investigated the effects of three critical factors—solid-to-liquid ratio, crystallization temperature, and crystallization time—on NaX-type molecular sieve synthesis using an orthogonal experimental design. The relative significance of these factors was evaluated based on specific surface area measurements from nine orthogonal trials, with R-value analysis determining factor prioritization. The R-value reflects the magnitude of parameter influence, where higher values indicate a greater factor dominance in terms of affecting synthesis outcomes. A data analysis identified the optimal synthesis conditions, as detailed in Table 4.

Using a specific surface area as the evaluation metric, range analysis (R-value method) revealed that the solid-to-liquid ratio and crystallization temperature exerted dominant effects on synthesis outcomes, while crystallization time showed minimal influence. ANOVA of the nine experimental groups further validated these findings: the calculated F-values for the solid-to-liquid ratio (F = 63.285) and temperature (F = 29.324) significantly exceeded the critical F-value (F<sub>crit</sub> = 19.000, α = 0.05), confirming their statistical significance. The consistency between ANOVA and range analysis conclusively demonstrated that synthesis performance is predominantly governed by the solid-to-liquid ratio and crystallization temperature. Table 5 provides detailed analysis data.

### 3.3. Characterization of Orthogonal Experimental Results

#### 3.3.1. XRD Characterization

**Figure 4:**
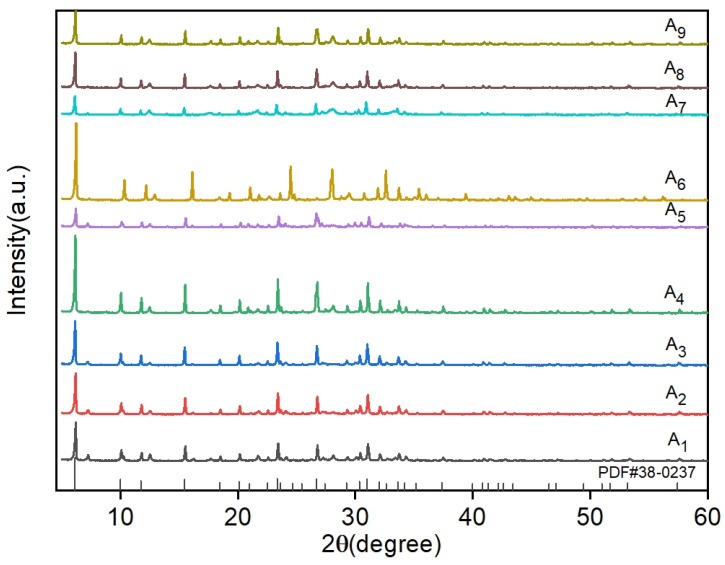
A_1_~A_9_ Comparative analysis of XRD results of nine groups of orthogonal experiments.

To systematically evaluate the effects of three critical parameters, i.e., solid-to-liquid ratio, crystallization time, and crystallization temperature, X-ray diffraction (XRD) analysis was conducted on nine orthogonal experimental groups. The acquired diffraction patterns were rigorously compared with the standard NaX-type molecular sieve reference (ICDD PDF#38-0237). A detailed phase composition analysis and crystallinity comparisons across all experimental conditions are comprehensively presented in Figure 4.

A comparative XRD analysis of samples A_1_~A_9_ revealed distinct characteristic peaks at 2θ = 6.103°, 9.986°, 11.727°, 15.451°, 20.073°, 23.611°, 27.394°, 30.971°, and 32.006°, corresponding to the (111), (220), (311), (331), (440), (622), (731), (751), and (840) crystal planes of NaX-type molecular sieves (ICDD PDF#38-0237). Close alignment with reference diffraction patterns confirmed the successful synthesis of NaX-type molecular sieves across all nine orthogonal experiments. The observed intensity variations among samples reflected crystallinity and purity differences influenced by factor-level combinations. Notably, samples A4 and A6 exhibited maximal peak intensities and areas, with quantitative crystallinity calculations yielding 98.8%, consistent with their superior structural ordering. These results validate the orthogonal design’s ability to satisfy NaX nucleation requirements while highlighting the critical role of parameter optimization in crystallographic quality control.

Combined with the results of orthogonal experiments, it was determined that the solid-to-liquid ratio has a large influence on the synthesis of the target molecular sieves [36]. The solid-to-liquid ratio, which reflects solution alkalinity, critically determines the molecular sieve crystal morphology. This parameter regulates OH^−^ concentration, governing the dissolution efficiency of inert SiO_2_ and Al_2_O_3_ components in coal gangue and modulating the resultant SiO_2_/Al_2_O_3_ ratio. Excessively high OH^−^ concentrations (>13 pH) reduce silicate polymerization degrees, enhance the dissolution of inert silica-alumina phases, and elevate the system’s SiO_2_/Al_2_O_3_ ratio, thereby favoring low-silica-alumina sodalite crystallization. This mechanism aligns with the dissolution–polymerization equilibrium theory in zeolite hydrothermal synthesis, where the solid-to-liquid ratio controls precursor dissolution-polymerization dynamics via OH^−^ concentration modulation.

During the dissolution phase, high solid-to-liquid ratios (e.g., 1:4) induce localized hyperalkalinity (pH >13), cleaving Si-O-Si and Al-O-Al bonds to generate reactive SiO_4_⁴^−^ and AlO_2_^−^ monomers. In the nucleation phase, elevated OH^−^ concentrations accelerate silica-alumina monomer condensation into primary gel particles (<5 nm), with spontaneous nucleation rates surging upon reaching critical supersaturation thresholds. At the crystal growth stage, a limited liquid-phase volume under high ratios increases mass-transfer resistance, suppressing anisotropic crystal growth and yielding uniform nanocrystallites (evidenced by reduced XRD peak widths and >85% crystallinity).

Theoretical modeling correlates solid-to-liquid ratio dominance across three stages: solubility, nucleation rate, and crystal growth. Ratios exceeding 1:4 restrict silica-alumina monomer diffusion in gel phases, elevating nucleation density while curtailing growth rates to produce small, high-crystallinity sieves. Conversely, low ratios (e.g., 1:10) dilute OH^−^ concentrations, prolonging induction periods and promoting heterocrystalline phases such as P-type zeolites.

As can be seen in Figure 4, when the solid-to-liquid ratio is 1:6, the diffraction peaks of NaX-type zeolite molecular sieves are more complete. When the solid-to-liquid ratio increased to 1:8, the peak intensity gradually increased. Sample A_4_ and the standard value (ICDD PDF#38-0237) compared to the crystal peaks were consistent, with the characteristics of the peaks being sharp and complete, indicating that at this time, the concentration of the system was suitable for the growth of the target zeolite molecular sieve. When the solid-to-liquid ratio is further increased to 1:10, sample A_7_ showed the same crystalline peaks but the intensity of the diffraction peaks gradually decreased, which may have been due to the incomplete dissolution of the raw materials when the alkali concentration was too low and the initial gel generation was less, resulting in incomplete crystallization and lower crystallinity. Our analysis of the XRD patterns showed that structures such as NaAlSiO_4_ and calcite were formed.

Crystallization time moderately influences molecular sieve crystallinity and grain size, with extended durations generally promoting crystal growth. However, within the tested time range, this parameter exhibited minimal impact on synthesis outcomes. In contrast, crystallization temperature critically governs reaction energy: excessive temperatures degrade crystallinity and induce crystal fragmentation by altering the pressure dynamics in sealed reactors. Elevated temperatures increase internal pressure, disrupting nucleation kinetics and anisotropic crystal growth, ultimately modifying the structure of the final product.

#### 3.3.2. SEM Characterization

To assess crystallization completeness and identify intercrystalline defects, the surface morphology and crystal dimensions of synthesized molecular sieves were analyzed through scanning electron microscopy (SEM). All nine experimental samples were subjected to milling, sieving, and gold sputtering pretreatment prior to SEM characterization.

**Figure 5:**
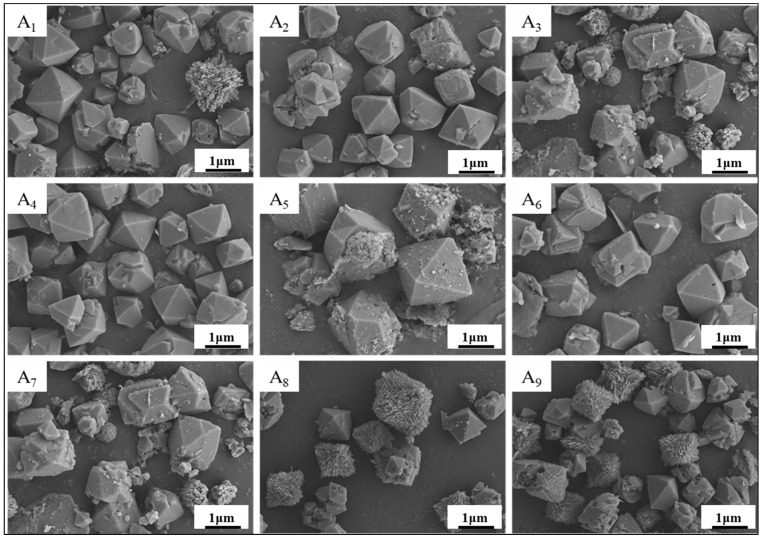
Electron micrographs of the results of orthogonal experiments of (**A_1_**)~(**A_9_**).

As shown in Figure 5, our SEM analysis confirmed that all nine samples exhibited the characteristic octahedral morphology of NaX-type molecular sieves, consistent with XRD phase identification. However, significant morphological variations were observed: certain samples displayed homogeneous octahedral crystals with well-defined facets (Figure 5A_4_), while others showed structural irregularities including fragmented crystallites (Figure 5A_7_), sporadic particle distributions, and incomplete octahedral formations. Notably, amorphous silica nanorods were detected in samples produced under suboptimal crystallization conditions. These microstructural disparities systematically correlate with orthogonal experimental parameters, demonstrating the critical influence of synthesis conditions on zeolite morphological evolution.

The solid-to-liquid ratio (alkalinity) of the system is the main influencing factor of this experiment. As can be seen from the electron microscope pictures, a low or high alkalinity affected the growth of the crystal particles; high alkalinity was conducive to the dissolution of silica-aluminum, but the crystal shape began to shift, and some of the crystals appeared defective and broken. Meanwhile, low alkalinity resulted not only the existence of sporadic octahedral structures, but also crystal shapes presenting irregular sphere structures, most of which existed in the form of nano-silica balls. Although there were sporadic octahedral structures with low alkalinity, the crystal morphology showed irregular spherical structures, mostly in the form of nano-silicon dioxide spheres, indicating that low alkalinity is not conducive to the nucleation of the NaX-type molecular sieves and the growth of crystals.

Comparatively speaking, the effect of crystallization time on the nucleation of NaX-type molecular sieves was relatively small. In conclusion, the SEM characterization results coincided with the XRD results, which also indicated that the experimental rule is universal, and the orthogonal experiments successfully supported this approach to synthesizing NaX-type molecular sieves, finding the optimal experimental conditions and yielding the best NaX-type molecular sieves. This finding laid the foundation for the subsequent adsorption experiments.

#### 3.3.3. Characterization of the Products Under the Best Conditions

**Figure 6:**
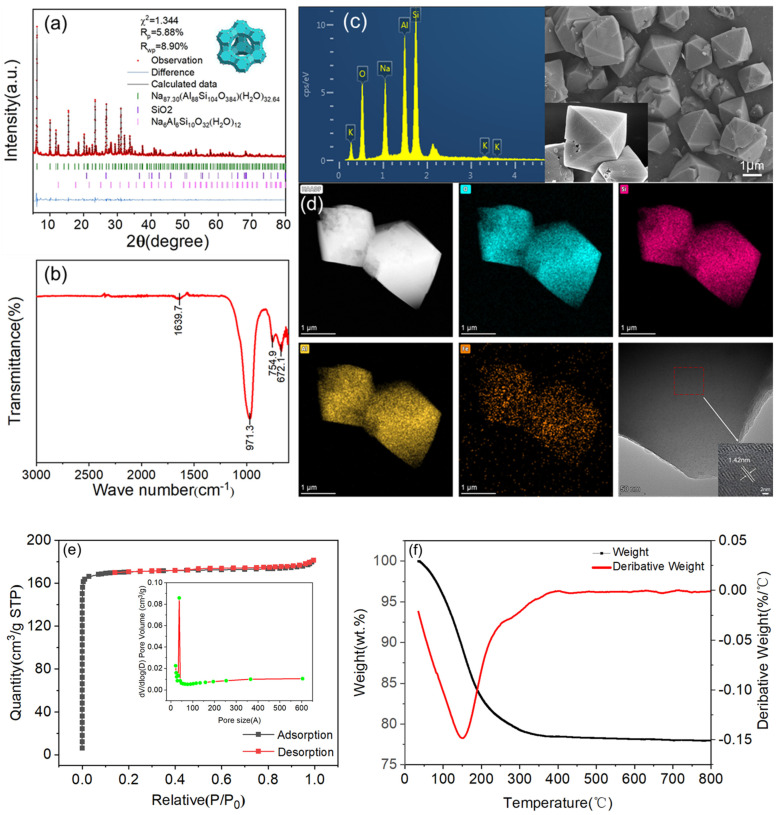
(**a**) NaX type molecular sieve XRD map refinement results; (**b**) sample FT−IR spectrum; (**c**) sample SEM−EDS map; (**d**) sample microstructure and elemental distribution of TEM map; (**e**) sample N_2_ adsorption and desorption isotherm (BET) map and the BJH pore size distribution; (**f**) the relationship between the best product TG−DTG curve.

Orthogonal experimental optimization identified the optimal synthesis parameters for NaX-type molecular sieves. High-performance molecular sieves were successfully synthesized from coal gangue under these conditions and subsequently selected for adsorption performance evaluations to assess practical applicability. The optimally synthesized products underwent comprehensive characterization to validate their structural integrity and functional properties. Detailed characterization results are shown in Figure 6.

The refined XRD pattern of the sample corresponded to the peak pattern of the standard (ICDD PDF#38-0237) card crystal surface, with fewer stray peaks and typical characteristic peaks that were sharp and complete. The difference between the calculated value and the fitted value was very small, which indicated that the product crystallization effect was good. The calculated results showed that the crystallinity was as high as 98.5% and the purity was 88.7%, indicating that NaX−type molecular sieves with high purity and performance were synthesized. The FT-IR spectra also showed peaks corresponding to those of industrial standard NaX-type molecular sieves at the peak positions of 1640.43 cm^−1^, 989.10 cm^−1^, 755.20 cm^−1^, 561.89 cm^−1^ and 481.38 cm^−1^. The infrared peaks corresponding to the industrial standard NaX-type molecular sieves all appeared, indicating that the two tetrahedra of SiO_4_ and AlO_4_ had been bridged by oxygen atoms, which further indicated that the synthesized product matched standard NaX-type molecular sieves. From the SEM picture, we can see that the NaX-type molecular sieve crystal morphology was a standard octahedral structure, and the crystal angles were clear, the dimensions homogeneous, and the crystal surfaces smooth and clean without defects. Also, the diameters of crystal particles were around 2 µm, which indicated that the crystal particles were about 2 µm. The diameter of the crystal particles was about 2 µm, which indicated that the synthesized product was successful. Meanwhile, an EDS spectroscopy analysis showed that the sample contained elements such as Si, Al, O, and Na, and the ratio of silicon to aluminum was about 1.3, which is in line with the theoretical value. A transmission electron microscopy (TEM) picture showed that (1) the microcrystal structure of the sample matched the SEM results, (2) the main elements, i.e., O, Al, and Si, were uniformly distributed, (3) a small amount of Fe and Ti were present in the sample, and (4) obvious lattice stripes of 20 nm in size were present.

The N_2_ adsorption–desorption isotherm (BET) diagram of the sample is a type I isotherm. The adsorption amount rose rapidly under the lower relative pressure, and the adsorption was saturated after reaching a certain relative pressure, which is the adsorption characteristic of microporous materials. It can be seen that the adsorption–desorption isotherm of this molecular sieve was similar to a typical Langmuir I curve, which reflected the phenomenon of the filling of the micropores on the molecular sieve; this is typical of a physical adsorption process. The adsorption–desorption isotherm was similar to a typical Langmuir I curve. The pore size distribution graph showed that the pore size of the molecular sieve was mainly concentrated at about 1.5 nm, and there was also a little mesopore distribution, which is in line with the theoretical value of standard NaX−type molecular sieves. The calculated specific surface area of the sample was 703.5341 m^2^/g, which is 54 times more than that of the original gangue (13.0143 m^2^/g). Its pore volume reached 0.2799 m^3^/g. From the above data, it can be seen that the sample had a high specific surface area and a large pore volume, and its adsorption conformation was dominated by physical adsorption. Finally, it was easy to regenerate adsorbent materials in a cyclic manner. From the TG−DTG curve analysis, it can be seen that in the interval of 100~250 °C, the mass loss of the sample was about 20%, which was mainly due to the loss of water in the sample, while in the interval of 250~800 °C, the mass of the sample was maintained at about 78%, which indicated that the material had a high thermal stability, and the application of the temperature range was wide, meaning that it can cope with a variety of conventional working conditions. In summary, a characterization analysis showed that the experimental synthesis of gangue-based X-type molecular sieves achieved excellent performance.

### 3.4. Analysis of Adsorption Results

#### 3.4.1. Study of Different Adsorption Conditions

In order to investigate the adsorption performance of the experimentally synthesized NaX-type molecular sieves, single-component adsorption experiments were carried out with heavy metal ions (Cu^2+^) and carbon dioxide gas, respectively. Considering that there may be many influencing factors, the adsorption capacity of the molecular sieves for copper ions was investigated using a one-factor-variable method, i.e., fixing the dosage of molecular sieves at 1 g/L and keeping the adsorption ambient temperature and pH unchanged.

**Figure 7:**
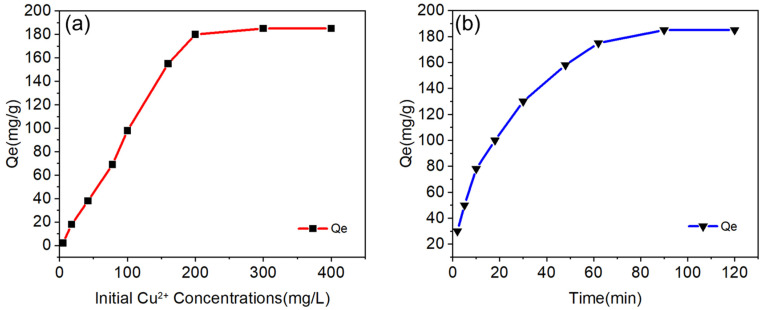

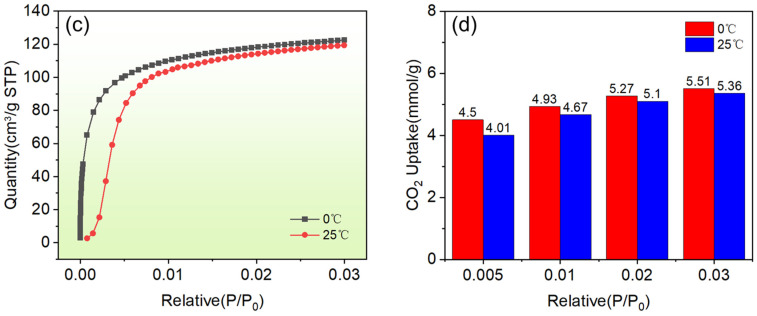
(**a**) Effect of initial solution (Cu^2+^) concentration on the adsorption performance (adsorption time: 1 h, adsorbent dosage 1 g/L, adsorption temperature: 293 K); (**b**) Effect of adsorption time on the adsorption performance (initial solution concentration of Cu^2+^: 200 mg/L, adsorbent dosage: 1 g/L, temperature: 293 K); (**c**) Effects of synthetic molecular sieves on the adsorption of Cu^2+^ at different temperatures and variable pressures. (**c**) Adsorption curves of synthetic molecular sieves on CO_2_ gas at different temperatures and pressures; (**d**) Histogram of the adsorption capacity of NaX-type molecular sieves on CO_2_ at different temperatures.

Figure 7a shows that the adsorption efficiency was close to 100% when the initial solution (Cu^2+^) concentration was low. With the increase of the initial solution concentration, the adsorption amount gradually tended to equilibrium, and when the equilibrium was reached, all the adsorption sites of the molecular sieve were saturated and the adsorption amount at the equilibrium was kept stable. After that, the adsorption capacity of the molecular sieve did not increase by continuing to increase the concentration of the initial solution. Therefore, the maximum adsorption capacity of the synthetic molecular sieve under this condition could be calculated from Equation (1): 185.35 mg·g^−1^ Subsequently, we chose an initial concentration of Cu^2+^ solution as 200 mg/L for the experiment. Figure 7b shows that the adsorption rate was very high in the first few minutes and the adsorption capacity tended to stabilize with the passage of time. When the adsorption time reached 1.5 h, the adsorption of Cu^2+^ by molecular sieves reached equilibrium. It can be seen that at the initial stage, there were sufficient active sites on the molecular sieves and an excess of adsorbate in the solution, so the adsorption efficiency was high [37]. With time, most of the adsorption sites were occupied and the adsorption rate slowly decreased, and the adsorption equilibrium was reached at about 1.5 h. Table 6 compares the adsorption capacity of different types of molecular sieves for Cu^2+^. The results show that the synthetic molecular sieves achieved excellent performance [38,39,40].

Figure 7c shows the adsorption capacity curves of molecular sieves for CO_2_ under variable pressure conditions, from which it can be seen that, in the initial stage, the adsorption capacity of the molecular sieve samples increased sharply with the rise of pressure. This is a typical adsorption characteristic of microporous-rich materials with many active sites in the initial pore channels which can fill with CO_2_ molecules quickly; an increase of pressure enhances the expansion and contraction vibration of the SiO_4_ and AlO_4_ backbones, positively promoting the further expansion of molecular sieve porosity. In addition, in the temperature range of 0~25 °C, the lower the temperature, the higher the adsorption capacity of the synthetic molecular sieves for CO_2_, which reflects the adsorption law of the gas to a certain extent. Figure 7d shows histograms of the CO_2_ adsorption capacity of the synthetic molecular sieves at several different pressure points with adsorption temperatures of 0 °C and 25 °C. It can be seen that the optimal adsorption capacities at 0 °C and 25 °C were 5.51 mmol/g and 5.36 mmol/g, respectively.

(1) $$Q_{e}=\frac{C_{0}-C_{e}}{W}\times V$$

where “*Q_e_*” is the equilibrium adsorption amount (mg/L), “*C*_0_” is the initial solution Cu^2+^ concentration, and “*C_e_*” is the solution concentration at equilibrium (mg/L). “*V*” is the volume of the solution (L), and “*W*” is the mass of the adsorbent molecular sieve (g).

#### 3.4.2. Adsorption Isotherm Studies

In this study, two common adsorption isotherms, Langmuir and Freundlich, were chosen to study the above adsorption test data. The equilibrium adsorption amount and equilibrium concentration of Cu^2+^ adsorbed on synthetic molecular sieves to reach adsorption equilibrium were fitted nonlinearly, and the best-fit correlation coefficients (R^2^) were evaluated and compared using linear form of isotherm equations. The Langmuir adsorption model assumes that adsorbates are piled up in a monolayer on the surface of the adsorbent, thereby forming the maximum adsorption amount. The molecules of the adsorbent are similar to those of an ideal gas, and adsorption and desorption are reversible processes. The adsorption energy is constant throughout the process and no migration of the adsorbate occurs at the surface [39,40]. The mathematical expression for this process is Equation (2). Equation (2) is mathematically processed and its linear expression is Equation (3).

(2) $$Q_{e}=Q_{m}\frac{K_{L}C_{e}}{1+K_{L}C_{e}}$$

(3) $$\frac{1}{Q_{e}}=\frac{1}{{Q_{m}K}_{L}}\times \frac{1}{C_{e}}+\frac{1}{Q_{m}}$$

where “*Q_m_*” (mg/g) is the monolayer adsorption capacity (maximum) and “*K_L_*” (L/mg) is the Langmuir isotherm constant. The Freundlich isotherm model is an empirical equation that is commonly used to describe the adsorption constants of nonpolar substances in gases and solutions. It is based on the assumption that adsorption occurs on the surface of the adsorbent through a multilayer adsorption mechanism; its isotherm is mathematically represented in Equation (4). The mathematical expression after linear treatment is Equation (5).

(4) $$Q_{e}=K_{f}{C_{e}}^{\left(1/n\right)}$$

(5) $$\log Q_{e}=\log K_{f}+(1/n)\log C_{e}$$

**Figure 8:**
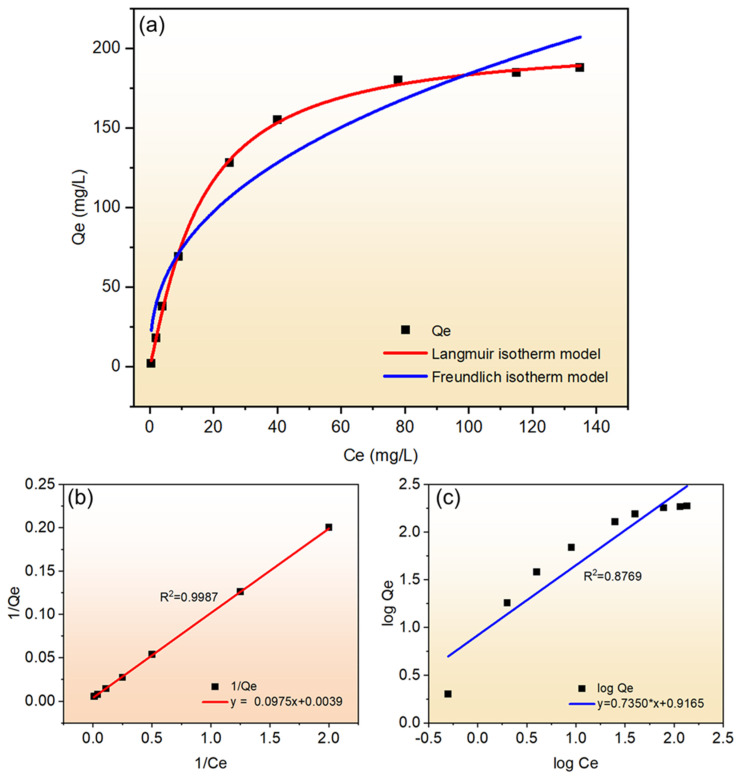
(**a**) Langmuir and Freundlich isotherm model nonlinear fit curves; (**b**) Langmuir model linear fit curve; (**c**) Freundlich model linear fit curve.

“*K_f_*” (mg/g) is the Freundlich constant, (1/*n*) denotes the adsorption strength, “*K_f_*” and “*n*” can be obtained from the intercept and slope of the linear plot of log *Q_e_* versus log *C_e_*. “*K_f_*” is used to denote the adsorption capacity, while (1/*n*) denotes the ease of adsorption during the adsorption process, which is classified as irreversible adsorption (1/*n* = 0), favorable adsorption (0 < 1/*n* < 1), and unfavorable adsorption (1/*n* > 1). For Cu^2+^, the fitted curves of the two adsorption models are shown in Figure 8.

As can be seen from Figure 8a, comparing the two curves, all the data points fell near the Langmuir adsorption isotherm model, and the R^2^ value of the linear fit to it was 0.9987, which indicated that the adsorption of Cu^2+^ from solution by the synthetic molecular sieves was more in accordance with the Langmuir adsorption model. As can be seen in Figure 8b, the value of 1/*Q_e_* was found to be increasing linearly with the value of 1/C_e_ in the concentration-corresponding part after fitting the data points. Based on the adsorption test for Cu^2+^, the R^2^ (0.9987) value of the linear fit was greater than 0.99, which also confirmed the pattern demonstrated in Figure 8a and proved that the adsorption process of Cu^2+^ by the synthetic molecular sieves was a monolayer adsorption. In contrast, the Freundlich isotherm model fitted poorly and the linear fitting result of R^2^ (0.8769) was also less than 0.99, indicating that the Freundlich model could not explain the adsorption law of these synthetic molecular sieves for Cu^2+^. Although the Langmuir model (R^2^ = 0.9987) in this study was able to describe the Cu^2+^ adsorption equilibrium tentatively, its monolayer adsorption assumption did not adequately reflect the stoichiometric properties of ion exchange. A subsequent combination of Na^+^ release data with the modified Sips model could more accurately characterize the adsorption process with multi-mechanism synergy.

To elucidate the CO_2_ adsorption behavior, the BET model was employed for mechanistic interpretation. As shown in Figure 7c, the variable-pressure adsorption isotherm resembled a Langmuir-type profile, exhibiting rapid uptake at low pressures followed by equilibrium saturation. However, the Langmuir model failed to describe CO_2_’s multilayer physisorption and micropore-filling effects within the NaX molecular sieves. Experimental isotherms revealed a steep adsorption rise in the low-pressure regime (P/P_0_ < 0.1), characteristic of Type I microporous filling, with adsorption kinetics governed by CO_2_’s kinetic diameter (0.33 nm). The subsequent slope reduction in the mid-pressure range (0.1 < P/P_0_ < 0.3) aligned with BET multilayer adsorption theory, reflecting density-gradient transitions of molecular arrangements within micropores.

The BET model thus delineated CO_2_’s multiscale adsorption mechanisms on the NaX sieves: cation site-driven monolayer adsorption dominated at low pressures, while quasi-multilayer adsorption occurred via van der Waals interactions in mid-pressure regions. Integration with microporosity correction methods (e.g., t-plot analysis) enabled a comprehensive characterization to be undertaken of these hierarchical adsorption processes, establishing theoretical guidelines for designing high-efficiency CO_2_ adsorbents with optimized pore-cation synergies [41,42].

Furthermore, a comparative analysis of N_2_ adsorption–desorption isotherms (Figure 6e) revealed a superior CO_2_ adsorption capacity in the synthesized molecular sieves, attributable to their pore architecture and surface affinity for CO_2_ molecules. This selectivity arose from differential molecular interactions governed by CO_2_’s higher polarizability (1.95 × 10^−39^ C·m^2^/V) and quadrupole moment (−14.3 × 10^−40^ C·m^2^), contrasting with N_2_’s lower polarizability (1.76 × 10^−39^ C·m^2^/V) and quadrupole moment (−4.7 × 10^−40^ C·m^2^). The minimal N_2_ affinity reflected its smaller kinetic diameter (3.64 Å), enabling unrestricted diffusion through micropores without stable surface binding. Conversely, CO_2_ molecules (kinetic diameter: 3.30 Å) exhibited strong interactions with the pore walls via electrostatic and van der Waals forces, achieving effective confinement within the molecular sieve channels. These findings demonstrated the material’s intrinsic CO_2_/N_2_ separation potential, providing critical insights for designing energy-efficient gas-selective adsorption systems.

In practical applications, water molecules (polar) and CO_2_ (quadrupole moment) competitively adsorb onto NaX molecular sieve surfaces via hydrogen bonding and electrostatic interactions. The strong hydrophilicity of Na^+^ cations in NaX molecular sieves results in preferential pore and cationic site occupation by H_2_O under high humidity, thereby shielding CO_2_ adsorption active sites. Although gangue-derived NaX exhibits CO_2_ adsorption capacity comparable to 13X zeolite under dry conditions, its performance under humid environments requires further validation.

Future studies should focus on mitigating water competition through surface hydrophobic modifications (e.g., organosilane grafting) or heteroatom doping (e.g., partial Na^+^ replacement with Mg^2+^). These strategies could weaken H_2_O interactions while enhancing CO_2_ trapping efficiency in moisture-rich conditions, advancing the material’s applicability in real-world gas separation systems.

## 4. Conclusions

(1) In this study, NaX-type molecular sieves were synthesized by an alkali fusion hydrothermal method using gangue as the basic raw material. Orthogonal experiments were used to investigate the effects of three main factors, namely, the solid-to-liquid ratio, crystallization time, and crystallization temperature, on the experiments. The results of extreme variance showed that the solid-to-liquid ratio had the greatest effect on the results of the experiments, with the crystallization temperature and time being the next most influential, as proven by the analysis of variance (ANOVA), which also showed that the solid-to-liquid ratio was significant.

(2) The molecular sieves synthesized under the optimum conditions were subjected to multiple characterizations by XRD, SEM-EDS, TEM, FT-IR, TGA, and BET. It was demonstrated that the synthesized molecular sieves possessed a number of favorable properties, such as high specific surface area (703.5341 m^2^/g), large pore volume (0.2799 m^3^/g), high crystallinity (98%), high purity (87.7%), and so on. Finally, Cu^2+^ ion and CO_2_ gas adsorption experiments were carried out on the synthetic molecular sieves. The maximum adsorption amounts of the two systems were found to be 185.35 mg/g and 5.51 mmol/g, respectively. Nonlinear fitting of the experimental data with the Langmuir and Freundlich adsorption models was attempted, and it was found that the data points were more in line with Langmuir adsorption isotherm model. It was then inferred that the adsorption was more in accordance with Langmuir adsorption isotherm model, which led to speculation that adsorption occurred via a monolayer adsorption process of ion exchange.

(3) In the analysis of our CO_2_ adsorption results, temperature was found to be the main factor affecting the performance. The synthetic molecular sieves were found to have more affinity for CO_2_ molecules after comparing the adsorption results of N_2_. In conclusion, in this study, a NaX-type porous molecular sieve was successfully prepared for the adsorption of heavy metal Cu^2+^ and CO_2_ waste gas, which could not only solve the problem of environmental pollution caused by gangue stacking, but also reduce the production cost of commercial molecular sieves and provide a high value-added use of coal gangue.

This study provides a scalable pathway for the conversion of CG into high-performance adsorbents. Future research should focus on the optimization of gangue-based X-type molecular sieves and function enhancement, focusing on the combination of machine learning or response surface methods to establish a multi-factor coupling model to achieve the precise control of crystallization temperature, acid leaching conditions, and other parameters, and, at the same time, the development of microwave-assisted, ultrasonic enhancement of green synthesis technology to reduce energy consumption. However, it is necessary to solve the problem of difficult to control the purity of the product due to microwave heating at the same time.. Raw material pretreatment needs to break through the selective acid leaching, graded roasting, and other impurity control technology and improve the stability of the silicon and aluminum ratio to ensure the structural homogeneity of the molecular sieves. Functional design can be tailored to the needs of wastewater treatment or gas separation, and multi-stage pore and composite structures can be constructed through template agent modulation or magnetic Fe_3_O_4_ doping to enhance adsorption selectivity and regeneration performance. The environmental benefits should be quantified through a whole life cycle assessment of carbon reduction potential and may be combined with the dynamic regeneration model to verify the economy of large-scale application. Simultaneously, pilot studies should be carried out to optimize the mass transfer efficiency and heat field distribution. Basic research should be combined with in situ XRD, molecular dynamics simulation, etc. to reveal the mechanism of silica-aluminate reconfiguration in the activation-crystallization process of coal gangue and the path of influence of impurities, so as to provide theoretical support for performance enhancement and ultimately to promote the technology from the laboratory to industrial applications.

## Figures and Tables

**Table 1:** Orthogonal table of molecular sieves L_9_(3^4^) prepared from coal gangue.

Experiment	1	2	3
Considerations	Ratio	Times (h)	Temperature (°C)
1	1:6	8	90
2	1:6	12	110
3	1:6	14	130
4	1:8	8	110
5	1:8	12	130
6	1:8	14	90
7	1:10	8	130
8	1:10	12	90
9	1:10	14	110

**Table 2:** Table of gangue composition content (%).

Ingredient	SiO_2_	Al_2_O_3_	TiO_2_	Fe_2_O_3_	ZrO_2_	MgO	K_2_O	Na_2_O	Other
Contain	57.84	25.19	0.87	8.46	0.02	2.25	3.08	1.15	1.14

**Table 3:** Results of acid leaching treatment of coal gangue.

Analyte	Compound Formula	Original Content (%)	Acid Leaching Content (%)
Na	Na_2_O	1.15	0.967
Si	Si_2_O_3_	57.84	76.25
Al	Al_2_O_3_	25.19	17.25
Fe	Fe_2_O_3_	8.46	0.96
Mg	MgO	2.25	0.80
P	P_2_O_5_	0.21	0.03
S	SO_3_	0.12	0.03
K	K_2_O	3.08	2.47
Ca	CaO	0.45	0.04
Ti	TiO_2_	0.87	1.07
Mn	MnO	0.16	0.01
Zr	ZrO_2_	0.02	0.01

**Table 4:** Visual analysis table of synthesis results.

Experiment	1	2	3	4	
Considerations	Ratio	Times (h)	Temperature (°C)	Blank	BET (m^2^/g)
1	1:6	8	90	1	360.165
2	1:6	12	110	2	427.274
3	1:6	14	130	3	290.365
4	1:8	8	110	3	703.534
5	1:8	12	130	1	523.563
6	1:8	14	90	2	645.584
7	1:10	8	130	2	260.385
8	1:10	12	90	3	445.588
9	1:10	14	110	1	511.246
K1	359.268	441.361	483.779	464.991	—
K2	624.227	465.475	547.351	444.414	—
K3	405.740	482.398	358.104	479.829	—
R	264.959	41.037	189.247	35.415	—

**Table 5:** ANOVA table.

Considerations	β^2^	n	F_1_	F_2_	Significance
Ratio	120,099.602	2	63.285	19.000	Yes
Times	2551.904	2	1.345	19.000	No
Temperature	55,649.990	2	29.324	19.000	Yes
Inaccuracies	1897.77	2			

**Table 6:** Cu^2+^ adsorption capacity of different molecular sieves.

Type of Zeolite	Maximal Adsorption Capacity (mg/g)	Literature Sources
NaX zeolite	185.35	This work
NaA zeolite	74.96	*Crystals* **2024**
ZSM-5	118.34	*Chemosphere* **2023**
NaY zeolite	75.18	*Journal of Sol-Gel Science and Technology* **2024**

## Data Availability

The original contributions presented in this study are included in the article. Further inquiries can be directed to the corresponding authors.
